# Development and Testing of an Owner‐Reported Outcome Measure of Clinical Signs and Quality of Life in Dogs Treated With Chemotherapy

**DOI:** 10.1111/vco.70028

**Published:** 2025-11-07

**Authors:** Jenny Harris, Katie Sutton, Quentin Fournier, Jo Armes, Emma Ream, Nicholas Bacon

**Affiliations:** ^1^ Faculty of Health and Medical Sciences School of Health Sciences, University of Surrey Guildford UK; ^2^ Lumbry Park Veterinary Specialists Alton UK; ^3^ AURA Veterinary Guildford UK; ^4^ Faculty of Health and Medical Sciences, School of Veterinary Medicine University of Surrey Guildford UK

**Keywords:** adverse events monitoring, canine oncology, chemotherapy, client‐reported outcome measures, clinical signs, quality of life, veterinary palliative care

## Abstract

Cancer is a leading cause of mortality in older dogs. Despite the prevalence of chemotherapy in canine oncology, a good understanding of owners' observations of side effects and clinical signs in real time is still lacking. Owners' perceptions and reporting of clinical signs play an important role in monitoring a dog's condition during treatment and the use of digital owner‐reported outcome measures could prove efficient in tracking chemotherapy side effects in the home environment. This could improve care and draws inspiration from the human use of patient‐reported outcome measures in oncology. We aimed to develop and test a prototype digital measure for monitoring clinical signs and health‐related quality of life in dogs undergoing chemotherapy, designed to facilitate owner participation in monitoring and support veterinary care. A rapid literature review was conducted to identify existing measures and their methodological limitations. Items were generated based on the Veterinary Comparative Oncology Group‐Common Terminology Criteria for Adverse Events, existing client‐reported outcome measures, and expert veterinary opinion. Proof‐of‐Concept testing was performed with 29 dog owners with pets undergoing chemotherapy. Participants completed daily assessments of their dog's clinical signs and weekly quality of life surveys over a 21‐day period. A sub‐sample participated in cognitive interviews to assess content validity and acceptability. Descriptive statistics were used to assess clinical signs and quality of life scores. Internal consistency and item discrimination were evaluated, and qualitative data were analyzed thematically. High adherence was reported, with a median of 21 daily and 3 weekly assessments completed. Participants found the assessments acceptable and beneficial. Fatigue, polydipsia, and anorexia were the most frequently reported clinical signs. Dogs experienced a median of 3 different clinical signs. The quality‐of‐life scale showed good internal consistency (Cronbach's *α* = 0.84). Participants appreciated the daily assessments, found them easy to complete, and believed the measure could help improve monitoring and decision‐making during chemotherapy. The prototype tool, the Canine Cancer Outcome Measure (CAN‐COM), demonstrated feasibility and acceptability for use by owners in the home environment for dogs undergoing chemotherapy. With further refinement and validation, such a tool could improve the monitoring of adverse events and support decision‐making in veterinary oncology, enhancing the welfare of canine cancer patients.

## 1 Introduction

Cancer is the primary cause of mortality in dogs [1] with chemotherapy being a commonly considered treatment option for certain cases. Whilst chemotherapy is generally well‐tolerated by dogs, the prevalence and risk factors for adverse events (AEs) and severe adverse events (sAEs) in veterinary oncology are poorly understood [2]. A systematic review of studies published between 2008 and 2014 highlighted that only a minority (19%) had prospectively planned standardised assessments of likely clinical signs (i.e., observable or measurable indications) at predefined intervals [3], with others relying on the spontaneous identification of AEs. This, coupled with low statistical power, might have led to the underestimation of the extent of sAEs in dogs [3]. Although this evidence is now over a decade old it underscores the need for more recent and rigorous approaches.

Furthermore, inadequate management of AE during treatment can diminish dogs' quality of life (QoL, the subjective assessment of the dog's overall wellbeing), undermine the curative intent of therapy, and present life‐threatening risks [2]. For owners, the impact of AE can be emotionally distressing and lead to multiple veterinary visits, increased costs and contribute towards negative perceptions of chemotherapy [4]. Additionally, little is known about the dog‐human behavioural mechanisms that may prompt help‐seeking by owners if a dog becomes unwell on treatment [5]. Comparable to human cancer patients [6], assessments of clinical signs in dogs depend on owners noticing changes in usual behaviour, recognising these signs as important and potentially affecting QoL, and deciding to take action. This may involve reporting the signs during routine veterinary appointments sometime after the event, or seeking more urgent veterinary care. However, any uncertainty or misattribution by owners may lead to delays in reporting, causing unnecessary discomfort and distress for the dog and potentially resulting in poorer outcomes.

Owner‐perceived QoL is a vital determinant of decision‐making in veterinary oncology [4, 7, 8]. Measuring QoL and its components in dogs and cats has become the focus of several recently developed questionnaires across a range of conditions, including cancer [9, 10, 11, 12, 13, 14]. However, these instruments have primarily been designed as research or data collection tools, for example, to standardise measurement within the context of clinical trials [11] and generally in oncology their development has not adhered to standard practice in the field of psychometrics for new instrument development [5].

In human oncology, patient‐reported outcome measures (PROMs) are increasingly used to evaluate study endpoints and serve as clinical practice tools to provide safer, more responsive, and personalized oncology care [6]. Smartphone and/or web‐based symptom monitoring platforms, underpinned by PROMs, have been developed to monitor symptoms remotely on a daily [15] or weekly basis [16, 17]. These platforms often include built‐in alerting algorithms, tailored evidence‐based self‐care advice, and feedback loops to oncology professionals [15, 16, 17]. Recent high‐quality randomized controlled trials (RCTs) have demonstrated multiple benefits of remote symptom monitoring using PROMs, including improved symptom management, reduced toxicity, enhanced QoL, increased self‐efficacy, reduced anxiety, enhanced survival, and reduced healthcare costs [15, 16, 17, 18, 19]. In veterinary medicine, these questionnaires are often described as Client Reported Outcome Measures (CROMs) [20] and have the potential to transform care and reporting in clinical trials, especially if combined with real‐time remote clinical sign monitoring. However, such benefits have not yet been widely explored in veterinary practice generally, or in veterinary oncology specifically.

To address this gap, this study aimed to develop, and test proof‐of‐concept of a prototype digital CROM in accordance with best practice guidance for early phase development of new questionnaires [21, 22, 23, 24]. Specific objectives were (i) to generate a prototype CROM (list of items) to measure both the onset of new clinical signs as well as ongoing QoL, (ii) proof‐of‐concept test the use of the CROM to collect initial data on adherence to completion and data quality, and (iii) gain qualitative feedback on the content validity and acceptability of the CROM including identifying any issues in comprehension and response.

## 2 Methods

### 2.1 Item Generation

The conceptual content of the CROM was identified following a previously published rapid literature review to scope the content and methodological quality of existing CROMs [5]. To summarise, this included mapping existing CROM content to the major categories identified in the Veterinary Comparative Oncology Group‐Common Terminology Criteria for Adverse Events (VCOG‐CTAE) [25, 26], health‐related QoL and associated subjective behaviours. The review found considerable variability in the recall period, content validity, and methodological rigour of existing CROMs and that their psychometric properties were rarely evaluated. The review supported the need for a comprehensive clinical sign and QoL measure for dogs being treated with chemotherapy [5]. Consequently, items were generated based on the constructs measured in existing CROMs, VCOG‐CTAE criteria, veterinary opinion on relevance, comprehensiveness and comprehension (NB, QF) and our knowledge of how similar constructs have been operationalised in instruments used to collect PROMs. Additionally, all items were evaluated using a standardised questionnaire pretesting checklist [27]. The resulting prototype, named Canine Cancer Outcome Measure (CAN‐COM), comprised a daily clinical signs scale to measure changes in potential indicators of chemotherapy side effects that would be observable by non‐clinicians. It is important to note that the clinical signs recorded may reflect chemotherapy side effects, cancer‐related side effects, concurrent medications that are not cytotoxic, or be from unrelated conditions. Additionally, CAN‐COM included a weekly measure of health‐related functional and social/emotional QoL over the past 7 days (see the section Scales and item scoring for further details and Table 1).

**TABLE 1:** **Clinical signs and quality of life (QoL)**. Summary of the clinical signs assessed and components of QoL measure in the CAN‐COM.

Daily assessment	
Clinical domain^a^	Specific clinical sign assessed (if different)
1. Activity levels/fatigue	—
2. Appetite	2.1 Reduce/loss of appetite 2.2 Other concern (e.g., difficulty chewing/swallowing)
3. Fluid intake	3.1 Not drinking as much as normal. 3.2 More thirsty than normal. 3.3 Other concern for example, difficulty swallowing, coughing/regurgitation after drinking
4. Bowel habits	4.1 Diarrhoea 4.2 Constipation 4.3 Other concern for example, passing blood (stools may be bright red or dark red/black) or mucus
5. Vomiting	—
6. Laboured breathing	—
7. Pain	—
8. Other clinical signs	Free text comments entered

Weekly assessments	
Subjective QoL domain^b^	Specific items
**Functional**	Problems walking about the home/garden
	2 Problems walking about outside
	3 Energetic and lively
	4 Coat is in good condition
	5 Itchy skin (scratching, biting, or licking)
	6 Coat is thinning/patchy/shedding more hair than usual
	7 Seems weak (e.g., unsteady gait or wide stance)
	8 Sleeping more than usual or seems lethargic
	9 Lost weight
**Social/Emotional**	10 Responds to presence (e.g., happy to see me when I get home)
	11 Enjoying life
	12 Seems anxious or distressed (e.g., cautious, distressed, frightened, upset)
	13 Mood seems low or sad (e.g., down in the dumps, withdrawn)
	14 More bad days than good
	15 Seems to be him/herself
	16 Interacting with other dogs the same way they usually do
**Global rating**	Overall quality of life on a scale from Poor (0) to excellent (10)

^a^ If present severity rated Mild, Moderate, Severe.

^b^ Subjective QoL was rated on a 4‐point scale from Not at all to Very much.

### 2.2 Proof of Concept Testing

#### 2.2.1 Ethical Review and Reporting Standards

The study was reviewed and approved by the University of Surrey Research and Ethics Committee (reference FHMS 21‐22 247 EGA) and reporting adheres to the content validity standards for the COSMIN Checklists for Assessing Study Qualities [28].

#### 2.2.2 Recruitment and Procedures

Potentially eligible participants were dog owners identified and approached through a specialist oncology service at a private veterinary referral hospital (AURA Veterinary). Eligibility criteria included adults aged ≥ 18 years, who had a companion dog diagnosed with cancer and receiving maximum tolerated dose chemotherapy. Chemotherapy drugs were considered to be cytotoxic drugs interfering with the cell cycle. Chemotherapy protocols could involve single or multi‐agent regimens and be scheduled as weekly, two or three‐weekly cycles. Dogs receiving Tyrosine Kinase Inhibitors or metronomic chemotherapy were excluded due to possible differences in clinical sign or quality of life profiles which could affect validation and to ensure a more homogeneous study sample at this early stage in development. Prednisolone administration was allowed as part of some standard chemotherapy protocols used to treat lymphomas and mast cell tumours (MCTs) but not included if a sole agent. Additionally, dogs with concurrent conditions significantly affecting QoL (e.g., diabetes mellitus, congestive heart failure, etc.) were ineligible. Owners were required to be fluent in spoken and written English, capable of providing informed consent, as assessed by veterinary staff, and indicate they had access to an online device (e.g., smartphone, tablet, laptop, or PC) for completing surveys and participating in interviews. Interested individuals were contacted by the research team (University of Surrey) via email or telephone to address any questions about the study and confirm their participation. The study target was to enrol 30 participants and dogs. For feasibility studies this is sufficient to assess acceptability and usability, and allowed for collection of both qualitative and quantitative data, providing insights into owner engagement, preliminary estimates of tool reliability and initial patterns of data [29]. This was appropriate for an early stage study to ensure manageability while gathering valuable information to refine the tool for larger studies [30, 31].

#### 2.2.3 Online Survey Procedures and Consent

A link to the baseline Qualtrics online survey was emailed to participants. The welcome page included a link to the participant information sheet and collected online consent which had to be confirmed to proceed. The baseline survey collected information about the dog and their treatment, owner demographics, and an initial assessment of QoL. Following completion of this survey, the clinical signs scale was emailed daily for 21 days from day 1 post‐chemotherapy, to enable detailed data collection throughout the maximum planned cycle, and the QoL scale was sent weekly during the 21‐day period (days 7, Time (T)1; 14, T2; and 21, T3). Participants were asked to complete the surveys at a similar time each morning as a routine to encourage compliance (e.g., after morning walk). An automated survey reminder was sent to non‐responders for both the baseline and weekly QoL assessments. We did not send reminders for the daily questionnaire as this could have overburdened participants. To protect animal welfare, participants were informed that the standard of care applied throughout this study, and that if they were concerned about the health or wellbeing of their dog, they should contact the veterinary practice in the usual way.

### 2.3 Cognitive Interviews

A sub‐sample (*n* = 10) of participants was invited by the researcher (KS) to participate in a semi‐structured online cognitive interview (30 min duration, see Supplement 1 for topic guide) [32]. Cognitive interviews allow for systematic probing into how well the CAN‐COM content performs; provide understanding of respondents' thought processes when answering them and help ensure good content validity (i.e., each item is relevant, comprehended, comprehensive and measuring concepts it is intended to measure) [32] and their sensitivity to generate information that could initiate supportive care advice on future digital platforms. Interviews also elicited participants' views on their priorities for any future digital platform and home monitoring package that could be used in routine care. Interviews were recorded using Microsoft Teams and transcriptions were downloaded directly, checked and anonymised. NVIVO software [33] was used to facilitate data analysis and storage. Interview recordings and transcriptions were stored on the password‐protected university server. Recordings were deleted after transcription checking and analysis was complete.

### 2.4 Scales and Item Scoring

#### 2.4.1 Daily Clinical Signs Scale

To minimise participant burden, the online scale completion employed programmed survey logic. Participants were first asked to indicate the presence or absence of seven core clinical domains, identified through our previous review and the VCOG‐CTAE framework [5, 25, 26]. If a domain was reported as present, the survey automatically routed participants to an additional question requesting further details and severity rating of mild (1), moderate (2) or severe (3) [3, 34] with accompanying descriptive anchors (Table 1). Participants could also report up to five additional clinical signs not captured by the predefined domain using a free‐text field.

#### 2.4.2 Quality of Life Scale

This comprised 19 items (12 functional and 7 social and emotional QoL) developed based on our previous review [5] and rated on a scale from not at all (1) to very much (4) to mirror human measures [34, 35] and total score computed (maximum score 56). An additional global QoL item was rated from 0 (worst) to 10 (best).

### 2.5 Analysis

#### 2.5.1 Statistical Analysis

Descriptive statistics are presented for sample characteristics, completion rates and the newly developed clinical signs and QoL scales. The presence of clinical signs over the period of up to 21 days was assessed by computing the number of dogs with each clinical sign, mean (standard deviation) and median (inter quartile range) for each clinical sign, total number of all clinical signs and clinical signs affecting each dog. For any clinical signs affecting > 1 dog, their daily frequency and 3‐day moving average (i.e., an average based on 3 consecutive days, smoothing short‐term fluctuations to visualise trends) are presented over the 21‐day period to allow exploration of variability and trends.

For QoL, descriptive statistics for the subscales and total score are presented at baseline, 7, 14 and 21 days. Median change between measurements was calculated. Internal consistency (Cronbach's α and McDonald's Ω) and item discrimination (item‐to‐total correlations) were assessed. Pre‐established thresholds for interpretation of internal consistency (coefficients > 0.7 = acceptable and > 0.8 = good) and item‐to‐total correlations (*r* < 0.02 = very weak) were used [22]. All analysis was conducted in Stata v16.

#### 2.5.2 Qualitative Analysis

Interview transcripts were read repeatedly and checked against initial recordings to ensure an in‐depth understanding of the data. Participant comments relating to the survey were identified per item (by KS) and stored as separate codes to ensure a thorough, systematic approach to data review [36]. Emerging general themes from the interviews were generated (by KS). Once the initial analysis was completed, findings were exported from NVIVO into a ‘framework matrix’ to facilitate a clearer overview of the results and discussion (KS, JA, JH).

## 3 Results

### 3.1 Recruitment and Characteristics of Sample

**FIGURE 1:**
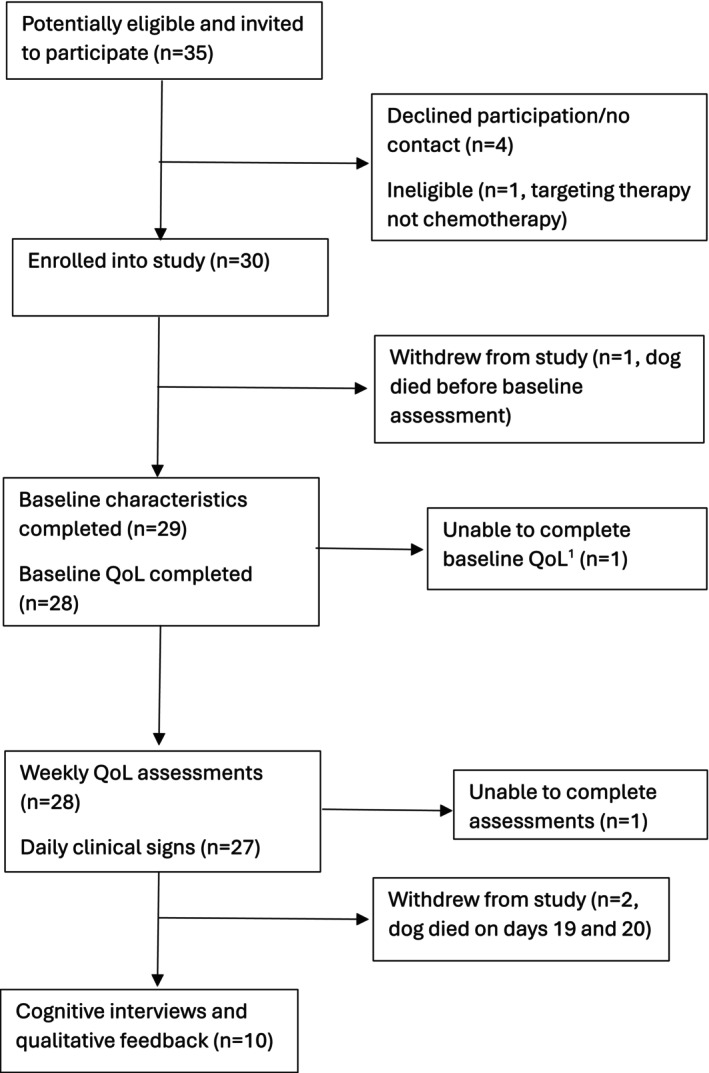
Study flow chart. Flow chart to show recruitment, withdrawal and participation in the study. ^1^Did not complete baseline QoL but completed subsequent QoL assessments.

Overall, 35 owners were approached to participate and 30 consented (Figure 1). One person had to unexpectedly withdraw after their dog's rapid deterioration before baseline assessment; one provided baseline characteristics but was unable to complete the baseline QoL assessment (although they participated in later weekly assessments), and one was lost to follow‐up due to technical issues. This resulted in 29 owners providing baseline characteristics, 28 providing baseline QoL, 28 completing weekly QoL assessments, and 27 completing daily clinical signs assessments. The owner sample had a median age of 50 (IQR 41–56), was mostly women (*n* = 20, 71%) and all described themselves as the primary or joint carer for their companion dog (Table 2). Most were White British or Irish (*n* = 25, 89%), educated to at least degree level or equivalent (*n* = 21, 75%) and two‐thirds were either employed or self‐employed (*n* = 18, 64.3%) (Table 2).

**TABLE 2:** Summary of sample baseline characteristics (*n* = 29). The baseline clinical characteristics of dogs and demographics of owners recruited into the study.

Clinical characteristics and demographics^a^	*n* (%) or Mean (SD)	Median (IQR)
*Companion dogs*		
*Age* (*years*)	8.3 (3.40)	*9.0 (6.0, 10.0)*
Sex status		
Female neutered	10 (33.3)	—
Male neutered	14 (46.7)	—
Male entire	5 (16.7)	—
Breed type		
Pedigree	24 (82.8)	—
Crossbreed	5 (17.2)	—
***Weight (kg)***	21.6 (13.39)	*18.3* (*11.5*, *31.5*)
Primary cancer diagnosis		
Mast cell tumours	11 (37.9)	—
Lymphomas	11 (37.9)	—
Sarcomas	5 (17.2)	—
Other	2 (6.9)	—
Chemotherapy treatment type		
Vinblastine	12 (41.4)	—
CHOP	6 (20.7)	—
Lomustine	3 (10.3)	—
Doxorubicin	3 (10.3)	—
Other	5 (17.2)	—
*Carer characteristics*		
Gender		
Male	8 (28.6)	—
Female	20 (71.4)	—
*Missing*	*1*	
*Age* (*years*)	48.0 (26.6)	*50.0 (41.0, 55.8)*
Relationship status		
Single/never married	3 (10.7)	—
Married or living with partner	18 (64.3)	—
In a relationship	2 (7.1)	—
Divorced or separated	5 (17.9)	—
Ethnic background		
White British or Irish	16 (53.3)	—
Any other white background	9 (30.0)	—
Chinese	1 (3.3)	—
Other Asian background	1 (3.3)	—
Prefer not to say	2 (6.7)	—
UK region		
Southeast	21 (75.0)	—
London	3 (10.7)	—
Northwest	1 (3.6)	—
Southwest	2 (7.1)	—
Yorkshire and Humberside	1 (3.6)	—
*Unknown*	*1*	—
Highest education level		
Degree or higher degree or equivalent	21 (75.0)	—
A Level or equivalent	5 (17.9)	—
O Level or GCSE or equivalent	1 (3.6)	—
Other	1 (3.6)	—
Employment status		
Employed full‐time	12 (42.9)	—
Employed part‐time	2 (7.1)	—
Unemployed	1 (3.6)	—
Self‐employed	4 (14.3)	—
Full‐time homemaker	2 (7.1)	—
Retired	5 (17.9)	—
Other	3 (10.7)	—
Carer for companion dog		
Client was main carer	18 (62.1)	—
Client was joint carer	10 (34.5)	—
Unknown	1 (3.4)	—

^a^ Values are presented as mean (SD) and median (IQR) for continuous variables, and *n* (%) for categorical variables.

At baseline, median dog age was 9 years (IQR, 6–10 years) and most were pedigree breeds (*n* = 24, 83%), of which there was a range of 21 Kennel Club recognised breeds. In total, 14 (48%) were male neutered, 10 (33%) female neutered and 5 (17%) male entire (Table 2). Cancer diagnosis included MCTs (11, 37.9%), lymphoma (11, 37.9%), sarcomas (5, 17.2%) and other tumour types (2, 6.9%). In terms of chemotherapy drugs/regimens, 12 (41%) dogs were treated with Vinblastine, 6 (21%) with CHOP (Cyclophosphamide, Vincristine, Doxorubicin and Prednisolone), 3 (10%) each with Doxorubicin or Lomustine, and 5 (17%) with other drugs including Vincristine and modified regimens. Chemotherapy cycle at enrolment ranged from cycle 1 to 7. In addition, 17 dogs were prescribed prednisolone as part of standard CHOP and vinblastine protocols, with additional use in lomustine and other regimens (*n* = 4). Two participants withdrew from the study after baseline assessments due to their dog's disease progression and subsequent death (at 19 and 20 days) and one person was unable to complete the daily assessment due to technical issues (Figure 1). With consent, their data were retained up to the point of withdrawal from the study.

### 3.2 Adherence to Assessments

Overall, adherence to the daily CAN‐COM and weekly QoL measures was good (Table 3). The median number of daily CAN‐COMs completed was 21 (IQR 18–21) and the median number of QoL assessments was 3 (IQR 2–3), and 85% of people completed at least three‐quarters of scheduled assessments. There were no missing items for those who completed either CAN‐COM or QoL scales (two participants completed one additional, and one participant completed three additional QoL assessments). In total this resulted in 504 individual CAN‐COM and 75 QoL assessments.

**TABLE 3:** Adherence to assessments. Overall adherence to assessments including mean (standard deviation, SD), median (25th and 75th centile of interquartile range, IQR), number completed 75% and 100%.

Assessment type	Adherence indicators				
	Mean (SD) completed	Median (IQR) completed and *range*	N completed at least 75%^a^	N completed 100%^a^	Total N completed
Daily CAN‐COM	18.0 (5.03)	21 (17.8, 21.0) *0–21*	23/28	11/28	504
QoL	2.7 (0.95)	3 (2.0, 3.0) 0–4	24/28	19/28	75

^a^ Note, includes two dogs that died during the study.

### 3.3 Daily Assessment of Clinical Signs

**FIGURE 2:**
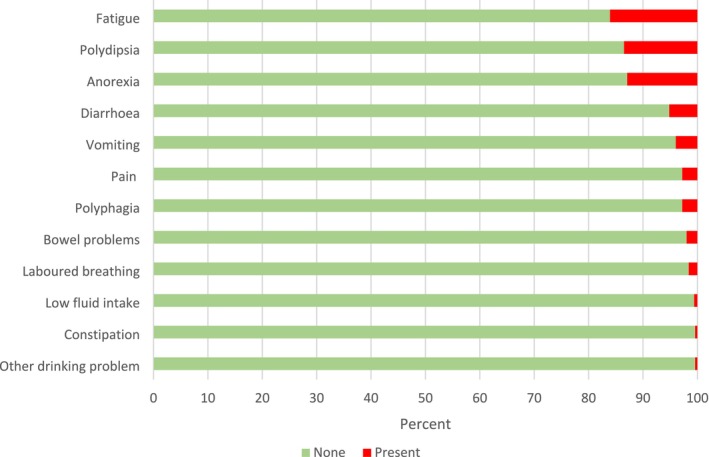
Clinical signs. Total occurrence of 12 clinical signs reported over the 21‐day period (*n* = 504 assessments). Red indicates sign was reported as present. Values are percents.

**FIGURE 3:**
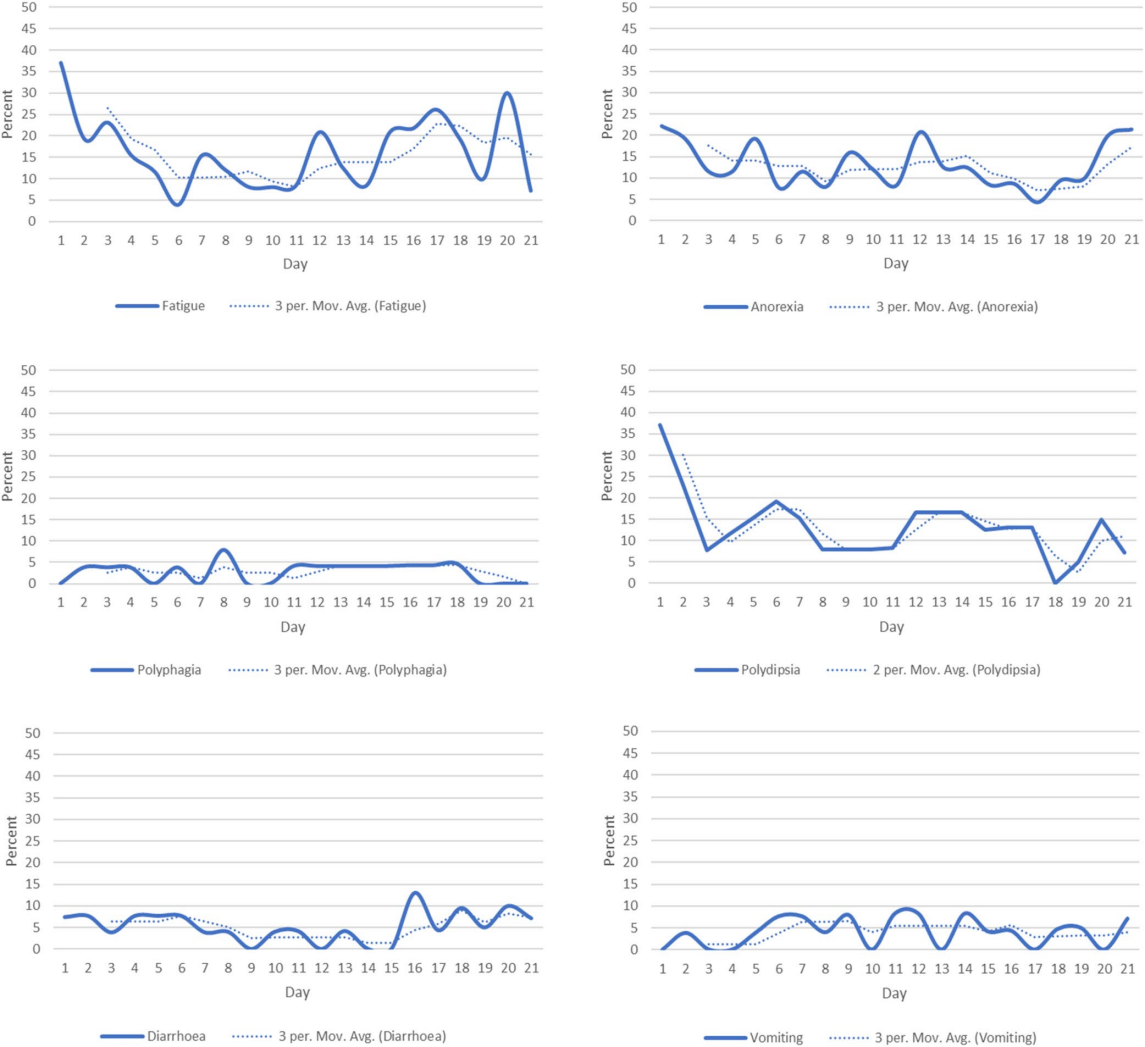
Percentage of clinical signs over 21‐day period. Daily (solid line) and 3‐day moving average (dotted line) of the percentage of sample with 6 clinical signs presented by observation day. Fatigue (top left); Anorexia (top right); Polyphagia (middle left); Polydipsia (middle right); Diarrhoea (bottom left); Vomiting (bottom right). Only clinical signs affected ≥ 2 dogs are presented.

Of all daily CAN‐COM assessments, over the 21‐day period, the most frequently reported signs were fatigue (16.1%), polydipsia (13.5%) and hyporexia/anorexia (12.9%), followed by diarrhoea (5.2%) and vomiting (4.0%) (Figure 2; and see Figure 3 for daily and 3‐day moving averages, Supporting Information 2). Less commonly reported signs were pain (2.8%), polyphagia (2.8%), constipation (< 1%) and other bowel problems (2.0%), and laboured breathing (1.6%). Low fluid intake and other drinking problems were reported less frequently (< 1% of signs). When experienced most clinical signs were evaluated as mild or moderate by owners (Supporting Information 3).

In terms of clinical signs by dog, 22/27 experienced fatigue, 14/27 polydipsia (including 13/21 dogs receiving prednisolone), 12/27 anorexia, 11/27 diarrhoea and 10/27 vomiting (Supporting Information 2). Dogs experienced a median of 3 different clinical signs over the 21 days, ranging from 1–11 (IQR 2–4). The mean total number of all types of clinical sign episodes experienced was 11.6 (SD 11.5) ranging from 1–48 episodes. Although some clinical signs were relatively uncommon, some individual dogs experienced repeated episodes. For example, dog 9 experienced a cluster of gastrointestinal signs including episodes of vomiting (4 episodes), diarrhoea (7 episodes) and other bowel problems (e.g., passing blood or mucus) (4 episodes), accompanied by reduced appetite (21 episodes) and constipation (2 episodes). It is notable that the dog with the greatest number of different types of clinical signs (11 different types) *and* individual clinical signs (48 episodes) died during the study period (dog 18, Supporting Information 2).

### 3.4 Quality of Life

Health related‐QoL total scores were negatively skewed; for example at baseline the mean score was 52 (SD 6.28; median 54, IQR 50–56) and ranged from 38–59 (Table 4). The two dogs who died during the study had total QoL scores that were lower than the 25th centile (scores of 38 and 41, out of a maximum of 76, Supporting Information 2). Median change QoL total score between baseline and T1 (7 days) was 3 (IQR1, 4) and varied from −7 to +16. Between T1 and T2 median change was 1 (IQR −1, 3) ranging from ±13.

**TABLE 4:** Descriptive statistics for CAN‐COM quality of life (QoL) scale. Descriptive statistics for QoL scale including mean (standard deviation, SD), median (25th and 75th centile of interquartile range, IQR) and range at baseline, day 7 (T1), day 14 (T2) and day 21 (T3). QoL items were rated on a 4‐point scale unless otherwise indicated.

Quality of life scales and individual item^1^	Baseline			T1 (day 7)			T2 (day 14)			T3 (day 21)		
	Mean (SD)	Median (IQR)	Range	Mean (SD)	Median (IQR)	Range	Mean (SD)	Median (IQR)	Range	Mean (SD)	Median (IQR)	Range
**Functional subscale (sum of items 1–9, max score 36)**	28.32 (3.61)	28.5 (25.5, 31.0)	19–33	29.56 (4.20)	30.0 (27.0, 33.0)	21–35	30.63 (3.31)	31.0 (29.0, 33.0)	21–35	29.74 (4.41)	30.0 (28.0,33.0)	17–35
Problems walking about the home/garden*	3.64 (0.73)	4.0 (3.5, 4.0)	1–4	2.74 (0.59)	3.0 (3.0,3.0)	1–3	2.71 (0.55)	3.0 (2.5, 3.0)	1–3	2.53 (0.70)	3.0 (2.0,3.0)	1–3
2 Problems walking about outside*	3.54 (0.79)	4.0 (3.0, 4.0)	1–4	3.56 (0.70)	4.0 (3.0,4.0)	2–4	3.67 (0.56)	4.0 (3.0,4.0)	2–4	3.37 (0.83)	3.0 (2.0,3.0)	1–4
3 Energetic and lively	2.89 (0.83)	3.0 (2.0, 3.5)	1–4	2.81 (0.92)	3.0 (2.0,3.0)	1–4	3.13 (0.68)	3.0 (3.0,4.0)	2–4	3.11 (0.88)	4.0 (3.0,4.0)	1–4
4 Condition of coat	3.43 (0.74)	4.0 (3.0, 4.0)	2–4	3.11 (1.01)	3.0 (2.0,4.0)	1–4	3.29 (0.91)	4.0 (3.0,4.0)	1–4	3.32 (0.89)	4.0 (3.0,4.0)	1–4
5 Itchy skin*	3.61 (0.74)	4.0 (3.0, 4.0)	1–4	3.56 (0.70)	4.0 (3.0,4.0)	2–4	3.58 (0.78)	4.0 (3.0,4.0)	1–4	3.68 (0.58)	4.0 (3.0,4.0)	2–4
6 Coat thinning/patchy/shedding*	1.96 (0.19)	2.0 (2.0, 2.0)	1–2	3.63 (0.88)	4.0 (4.0,4.0)	1–4	3.67 (0.64)	4.0 (3.5,4.0)	2–4	3.63 (0.76)	4.0 (3.0, 4.0)	1–4
7 Weak and unsteady gait or stance*	2.57 (0.93)	3.0 (2.0, 3.0)	1–3	3.48 (0.80)	4.0 (3.0,4.0)	1–4	3.71 (0.69)	4.0 (4.0,4.0)	1–4	3.37 (0.83)	4.0 (3.0,4.0)	1–4
8 Fatigue/lethargic*	3.29 (0.81)	3.0 (3.0, 4.0)	1–4	3.26 (0.81)	3.0 (3.0,4.0)	1–4	3.46 (0.66)	4.0 (3.0, 4.0)	2–4	3.32 (0.82)	3.0 (3.0,4.0)	1–4
9 Lost weight	3.39 (0.79)	4.0 (3.0, 4.0)	1–4	3.41 (0.89)	4.0 (3.0,4.0)	1–4	3.42 (0.93)	4.0 (3.0,4.0)	1–4	3.42 (0.96)	4.0 (3.0,4.0)	1–4
**Social & emotional subscale (sum of items 10–16, max score 28)**	23.54 (3.01)	24.5 (22.5, 26.0)	16–26	25.37 (4.12)	27.0 (24.0, 28.0)	12–28	25.58 (3.06)	27.0 (24.0, 28.0)	17–28	25.26 (4.45)	26.0 (25.0,28.0)	9–28
10 Responds to presence	3.89 (0.31)	4.0 (4.0, 4.0)	3–4	3.89 (0.42)	4.0 (4.0,4.0)	2–4	3.92 (0.28)	4.0 (4.0,4.0)	3–4	3.89 (0 0.32)	4.0 (4.0,4.0)	3–4
11 Enjoying life	3.64 (3.64)	4.0 (3.0, 4.0)	2–4	3.52 (0.80)	4.0 (3.0,4.0)	1–4	3.58 (0.58)	4.0 (3.0,4.0)	2–4	3.53 (0.77)	4.0 (3.0, 4.0)	1–4
12 Mood anxious or distressed*	2.75 (0.52)	3.0 (3.0, 3.0)	1–3	3.89 (0.42)	4.0 (4.0,4.0)	2–4	3.92 (0.28)	4.0 (4.0,4.0)	3–4	3.79 (0.71)	4.0 (4.0, 4.0)	1–4
13 Mood low or sad *	3.57 (0.74)	4.0 (3.0, 4.0)	1–4	3.59 (0.80)	4.0 (3.0,4.0)	1–4	3.67 (0.64)	4.0 (3.5,4.0)	2–4	3.74 (0.73)	4.0 (4.0, 4.0)	1–4
14 More bad days than good*	2.82 (0.48)	3.0 (3.0, 3.0)	1–3	3.41 (0.84)	4.0 (4.0,4.0)	1–4		4.0 (4.0,4.0)		3.79 (0.71)	4.0 (4.0, 4.0)	1–4
15 Like their usual self	3.36 (0.95)	4.0 (3.0,4.0)	1–4	3.37 (0.88)	4.0 (3.0,4.0)	1–4	3.21 (1.06)	4.0 (4.0,4.0)	1–4	3.21 (0.98)	4.0 (3.0, 4.0)	1–4
16 Interacting with other dogs	3.50 (0.84)	4.0 (3.0, 4.0)	1–4	3.89 (0.42)	4.0 (3.0,4.0)		3.50 (0.83)	4.0 (4.0,4.0)	1–4	3.32 (1.00)	4.0 (3.0, 4.0)	1–4
**Quality of life total score** ^**2**^	51.86 (6.09)	54.0 (49.5, 56.0)	38–59	54.93 (7.50)	56.0 (53.0, 60.0)	36–63	56.21 (5.28)	58.0 (53.0–63.0)	43–63	55.0 (8.32)	56.0 (52.0,61.0)	26–63
17 Global item rating overall quality of life on a scale 0–10^3^	8.1 (1.59)	8.0 (7.5, 9.0)	4–10	8.0 (2.02)	8.0 (7.0,9.0)	2–10	8.0 (1.53)	8.0 (7.5, 9.0)	4–10	7.95 (1.96)	9.0 (7.0, 9.0)	1–10

^1^ Specific items wording is shortened to broad topic area. Items rated on a scale from Not at all [1] to Very Much [4]. Higher score = better QoL.

^2^ Total sum of items 1–16, min/max score = 16–64.

^3^ Rated on an 11‐point scale from 0 (worse) to 10 (best).

* Indicates items reverse coded prior to analysis.

### 3.5 Internal Reliability

Internal consistency of the QoL scale was high (Cronbach's α 0.84, ranging 0.82–0.85 if an individual item was removed from the scale) (Supporting Information 4). Overall item discrimination for this scale supports the ability of individual items to discriminate between those with high or low overall scores, indicated by item to total correlation ranging from r 0.19 to 0.66. However, notably one item (coat thinning/patchy/shedding) had only a weak correlation (*r* = −0.07). The two subscales, functional and social/emotional, had good internal consistency (Cronbach's *α* 0.72 and 0.76, respectively) indicating the individual items within each subscale are measuring the same underlying constructs, and were moderately correlated (*r* = 0.69) which indicates that the subscales are measuring related yet distinct constructs.

### 3.6 Qualitative Feedback

Feedback from the cognitive interviews suggested that the assessments were highly acceptable to participants (Table 5) and several people spontaneously noted that the daily assessments were much less of a time commitment than they had originally anticipated. Generally, people found the assessments reassuring and they did not feel that completing them provoked worry or concern. One participant spontaneously mentioned that she had used the assessment to ask about her dogs' clinical signs in a later consultation: “In fact what happened was anything that came up that I thought about in the questionnaire I would just simply ask about the next time we came here, so if anything, the vet would put us back at ease right? And just made us feel better about it. Like for example the panting or his tiredness, they were like, yeah, this is totally normal. This is totally expected.”

**TABLE 5:** Summary of qualitative feedback. Qualitative theme, sub‐themes and example quotes based on interviews (*n* = 10).

Theme	Sub‐theme	Example quote
General comments		
		“Very easy… and quite helpful”
	Ease of use/understanding/usefulness	“The wording all makes perfect sense to me and it's very easy to understand… anything that came up that I thought about in the questionnaire I would just simply ask about the next time we came here, so if anything, the vet would put us back at ease”
		“It worked really well on my phone. It worked really well on my computer, which is a big thing. Everything's responsive as well and I didn't have any issues” “It didn't take long”
	Frequency/timing of collection	“[Filling it in everyday] it was easy”
		“I think the timing that you sent it out was perfect. I think that was a good time to send it out and was a good reminder to me to do it at that point in time”
		“It's absolutely fine [completing questionnaire everyday]. It is absolutely fine. It's good that it comes through at 8 am, so I can just click on it and get it done”
	Rating scale	“Very good. And I think I normally put him as about 9 out of 10 in general quality of life. That's very easy and quick to do”
		“Yeah, that's fine. Yeah, well, that is fairly standard, isn't it? And that's nice. Easy way of doing it”
Areas for development	Time period of assessment	“I found when I was answering the questions. I had to remind myself with each question that you're asking about how the dog was feeling today versus the day before. Instead of today versus before chemotherapy started” ‘My dog's appetite has changed’. Again. It's a clear question, but I struggled with it, you know. No, it hasn't changed since chemo started. Yes, it has changed significantly since before chemo started” “I really struggled with this because it felt like important for someone to know that she was drinking a lot more than before the chemo, but again, it wasn't really what you were asking. You were asking within the chemo process. Was it changing? “
		“I suppose it's just whether you're saying you know, today is she drinking less than she was before she was poorly or before chemo? Or so it's kind of what the. So, although it is in what it is today, it's what's what is it the reflection of? Is it reflection of? Compared to last week? Or you know what? What? What's the kind of measurement against?”
	Difficulties responding when have existing conditions and so forth.	“He's not very active anyway. And his breed as well. So, I guess it's just a point of saying the dogs who have had legs amputated, they're not very active anyway…and therefore it's very sort of difficult to say whether he's less active on a daily basis as he's just a rug on the floor anyway. That's all he ever does. So, it might be better to have a little bit of wording in you know that is sort of more about is he less active than he used to be…before starting chemo, if that makes sense”
	Attributing changes to other reasons	“He was on steroids. So, he's just incredibly hungry all the time”
		“Diarrhoea Can also be for like 1000 reasons. Yeah, sometimes he eats just bones. Yeah, you're like, whatever. So, I'm just trying to think how much of it I'm thinking in relation to the chemotherapy”
	Assessing dogs experience	“I struggled a little bit… just as the owner trying to put myself in her position and her experience”
		“I just scored him as a seven, OK. The only reason I scored him a little lower is not from the chemo. It's more obviously after having the surgery”
Views on experience of using CAN‐COM, clinical signs/QoL during CT	Raised awareness/understanding	“He's been very, very well. Like, we're shocked at how well he's been”
		“I've been quite lucky. She's not been that poorly. She's been, really. She's done very well since she's been on the chemo, actually”
		“So apart from being a bit more lethargic, although these are these other ones just aren't going to apply to him cause he's just bouncing back”
		“(Before the study) I did think… actually the information sheet did note that you may feel more concerned about your dog's wellbeing because you're recalling it in detail every day. But actually, I didn't, in fact”
	Reassurance	“The reason that I said that we would do it is because my mum was dead against us doing chemo, OK? Absolutely, completely, very vocal about it. And like, why are you putting them through that? And I think, I was a little bit concerned about putting him through it, but my husband was like nope we're doing it, it is fine. And it's helping to change those perspectives…For the people like my mum that was, you know, why would you do that? … I'm completely shocked with how well he's doing. So, it's been really helpful to fill out the questionnaire and take part in the study”
	Reflect on concerns	“I'd say that's what this questionnaire sort of perhaps did. It made you look at things a little bit more in the sense of the emotional side of it. Am I doing the right thing for him?”

Another person mentioned that completing CAN‐COM had reassured her about the decision to use chemotherapy for her dog (Table 4). Only one person mentioned that completing CAN‐COM might have made them focus more on the emotional experiences of chemotherapy and more deeply consider their decision making around choosing chemotherapy; “it leads into more questions about me as a dog carer if I am doing the right thing by putting him through this.”

The interviews suggested that people found the items relevant, easy to understand and answer (Table 4). Five participants said, for the daily clinical signs assessment, that they sometimes found it difficult to remember the correct reference period, specifically, if they were meant to be answering about whether their dogs' clinical signs had changed compared to the last assessment (i.e., the day before) or if the comparison was to their pre‐chemotherapy health state. They agreed that a clear anchoring statement reminding them of the reference period on each item would help with this recall. Most thought that the CAN‐COM could generate information that they could discuss with their vet and consequently better understand and support their dogs' wellbeing during chemotherapy.

## 4 Discussion

This study is amongst the first to develop and test a digital CROM specifically for dogs undergoing chemotherapy, adhering to best practice for content validity for scale development [23, 28], and providing novel insights into the feasibility of remote monitoring of clinical signs and QoL in veterinary oncology. Our findings demonstrate the feasibility and high adherence to a digital, daily CAN‐COM, with most participants completing the required assessments. This suggests that daily monitoring is not overly burdensome and, with further validation, may be a valuable tool for collecting real‐time data on dogs' clinical signs and health‐related QoL during chemotherapy treatment.

The most frequently reported clinical signs in our study were fatigue, polydipsia, and anorexia, followed by gastrointestinal signs such as diarrhoea and vomiting. These findings are consistent with previous reports on the chemotherapy‐associated AEs in dogs [2, 37, 38]. However, it should be noted that prednisolone was permitted as part of standard therapy in some dogs with lymphomas and MCTs and, given its well‐recognised side effect profile [39], concurrent prednisolone use may have contributed to the high frequency of polydipsia observed. However, our study adds novel insight by identifying preliminary evidence of the variability in the number and frequency of clinical signs. For example, some dogs experienced clusters of AEs, particularly gastrointestinal, which aligns with prior studies in human oncology where gastrointestinal toxicities are common [40], and in dog studies that have been noted as particularly influencing owners' perceptions of chemotherapy compared to other clinical signs [38].

Importantly, such monitoring approaches may serve as valuable research tools as we advance our understanding of factors influencing susceptibility to chemotherapy toxicities. Proposed determinants of drug tolerance include age, sex, obesity, organ function and genetic makeup. In humans, polymorphisms in genes involved in drug metabolism and regulation have been linked to chemotherapy sensitivity [41], with recent mouse studies suggesting around 50% of variability in response to anthracyclines [42]. In dogs, the only relevant genetic polymorphism identified so far is the MDR‐1 (ABCB1) mutation, typically found in Collie breeds, which is the only well‐characterised genetic variant associated with heightened sensitivity to P‐glycoprotein substrates such as anthracyclines and vinca alkaloids [43, 44, 45, 46, 47, 48]. As research evolves, especially in areas such as host genetics and gut microbiota [49, 50], our tool could support efforts to identify biomarkers or predictive profiles for chemotherapy tolerance and toxicity in canine patients.

Qualitative interviews indicated that whilst daily reporting was acceptable to owners and for some reassuring, a minority found it difficult to remember the reference period that they needed to consider when completing assessments, specifically whether they should be reporting changes in their dog prior to the start of chemotherapy or if it was changes since their last assessment (usually the day before). Therefore, future versions of the daily clinical signs CAN‐COM should improve the wording clarity and include reminders about the reference period throughout the survey (i.e., not only at the start of the CAN‐COM) [51]. This illustrates the importance of qualitatively evaluating new measures, alongside quantitative assessments, to ensure they are comprehended by the public and refined based on user feedback. Future research should test the predictive validity of such assessments [22] but might indicate that frequent monitoring of clinical signs could serve as an early warning system, prompting timely interventions that may mitigate AEs.

The distribution observed in the baseline QoL scores indicates that most owners perceived their dogs to have a relatively high QoL at the start of the study. However, two dogs with lower QoL scores were those that later died, suggesting that the QoL CAN‐COM may be sensitive enough to detect deteriorating health conditions in companion animals. QoL scores over time remained relatively stable for most dogs in our study and future larger scale research is needed to explore if this measure is sensitive enough to detect when dogs experience an early recovery or adaptation to treatment, or declining QoL due to treatment‐related toxicity or disease progression.

The internal reliability of our QoL scale was high, reinforcing the potential validity of this tool in assessing the broader impact of chemotherapy on dogs' functional and social–emotional wellbeing. Importantly, the qualitative feedback from owners underscored the practical utility of the CROM in supporting decision‐making, with some owners using the data to inform discussions with their veterinarians. This highlights the potential for CROMs to facilitate more informed, shared decision‐making between owners and veterinary professionals, similar to the role of patient‐reported outcome measures (PROMs) in human oncology [6].

The results of this proof‐of‐concept study suggest that integrating digital CROMs into routine canine oncology practice could offer several benefits. First, it would enable a more systematic and comprehensive approach to monitoring clinical signs and QoL in dogs undergoing chemotherapy [5]. Unlike spontaneous reporting of AEs, which may underreport the true burden of AEs [3], real‐time CROM monitoring provides a structured framework for capturing subtle changes in health status that could otherwise go unnoticed. This could improve early detection of AEs, allowing for timely interventions to prevent more severe complications. Additionally, long‐term there may be clinical utility in better understanding the range of clinical signs and QoL during treatment regardless of whether they are directly related to chemotherapy, the cancer or other conditions, as well as developing a ‘safety net’ of alerting algorithms to empower owners when to seek further veterinary support.

Second, incorporating CROMs could enhance owner communication and satisfaction [20]. By involving pet owners in the monitoring process, CROMs can empower people to play a more active role in their dog's care [20], potentially reducing anxiety and improving adherence to treatment plans. Our findings also suggest that participants appreciate the reassurance provided by CROMs, which may help alleviate the emotional distress in owners often associated with chemotherapy in companion animals [4].

### 4.1 Limitations

Although our study provides important insights, there are several limitations to consider. The sample size was relatively small, and the study was conducted over a limited time (21 days for each dog). Future research should aim to test CAN‐COM over longer durations and in larger canine populations with different types of cancer and chemotherapy regimens. Additionally, while our qualitative feedback suggests high acceptability of CAN‐COM, further research is needed to explore whether such tools could be effectively integrated into routine care settings.

A potential limitation of this study is the reliance on owner‐reported data, which may introduce subjectivity or bias. However, as often seen in human medicine, discrepancies exist between owners' and veterinarians' perceptions of QoL and clinical signs [20, 52]. For example, Scott et al. found that half of cat owners rated their cats as “perfectly healthy” despite a diagnosis of osteoarthritis, whereas veterinarians were more likely to recognise its impact on QoL [13]. Similarly, in human oncology, clinicians have been shown to underestimate the effects of treatment on QoL compared to patient‐reported outcomes [53, 54].

In human palliative care, the use of proxy assessments by family members or carers has been widely studied. While valuable, proxy evaluations often differ from patient self‐assessments, influenced by factors such as caregiver burden and the closeness of their relationship with the patient [55, 56]. In veterinary practice, where direct communication with patients is not possible, proxy reporting is essential and tools such as CROMs could serve as useful adjuncts in canine oncology. However, further research is needed to understand how owner assessments are shaped by their perceptions, emotional states and caregiving experiences. Refining the CAN‐COM in future studies will be essential to ensure it accurately measures both objective indicators of canine health and subjective aspects of QoL.

It should also be recognised that in this preliminary study it was not possible to determine if the clinical signs and quality of life results reflected chemotherapy side effects, cancer‐related side effects or unrelated conditions. It will be important for future studies to determine the likelihood that clinical signs were related to chemotherapy and to develop appropriate clinical pathways for when the CAN‐COM indicate a deterioration of health state.

## 5 Conclusion

This study demonstrates the feasibility and potential value of a digital CROM for monitoring clinical signs and QoL in dogs undergoing chemotherapy. The high adherence rates, coupled with the rich data on owner‐reported clinical signs and QoL, suggest that CAN‐COM could play an important role in optimising the management of clinical signs and supporting decision‐making in veterinary oncology. Further research is warranted to explore the broader application of these tools in clinical trials and routine veterinary practice and to assess their impact on clinical outcomes, owner satisfaction, and healthcare costs.

## Ethics Statement

The study was reviewed and approved by the University of Surrey's Research and Ethics Committee (reference FHMS 21‐22 247 EGA).

## Conflicts of Interest

The authors declare no conflicts of interest.

## Supporting information


**Data S1:** Supporting Information.

**Data S2:** Supporting Information.

## Data Availability

The data that support the findings of this study are available from the corresponding author upon reasonable request.
