# Supplementary material for: Development and Testing of an Owner‐Reported Outcome Measure of Clinical Signs and Quality of Life in Dogs Treated With Chemotherapy
Source: Vet Comp Oncol. 2025 Nov 7;24(1):80–94. doi: 10.1111/vco.70028 (PMC12875753; doi:10.1111/vco.70028)
Supplement: Supplementary file 1 — Data S1: Supporting Information. [file VCO-24-80-s001.docx]

Supplement 1

**Topic guide for cognitive interviews:**

| [Introduce yourself – check for questions based on PIS – check confidentiality understanding – take formal consent]   - Recap purpose of the questionnaire and the study - Trying to understand whether the draft questionnaire is easy to comprehend/ where errors may arise - Would like the participant to complete the questionnaire “out loud”, we will not be audio recording this but simply taking down some notes where questions may need some clarification etc.   There are no right or wrong answers, we are just interested in how the questionnaire works, whether questions are easy to understand/ complete and whether there are any misunderstandings in the way we have drafted the questions. |
| --- |

Interviewer to listen to participant complete questions in the draft survey during the interview and listen carefully to any potential confusion/ misunderstanding/ sources of error and note them down.

Potential prompts during survey:

- Does that make sense to you?
- What does that mean to you?
- Can you expand a little more on that?

At the end of the survey:

- How easy/ difficult did you find it to take part in this survey?
- Do you have any comments about the survey you would like to add?
- We are currently designing the future study and would like to find out if you thought the incentives we offered (shopping voucher) had any impact on your willingness to take part/continue with the study. Anything you would prefer (e.g. option to donate to an animal charity).
- We hope to conduct further research to develop a digital monitoring package based on the findings of this study. Would you be happy to be contacted about getting involved in a future project?
