# Supplementary material for: Development and Testing of an Owner‐Reported Outcome Measure of Clinical Signs and Quality of Life in Dogs Treated With Chemotherapy
Source: Vet Comp Oncol. 2025 Nov 7;24(1):80–94. doi: 10.1111/vco.70028 (PMC12875753; doi:10.1111/vco.70028)
Supplement: Supplementary file 2 — Data S2: Supporting Information. [file VCO-24-80-s002.docx]

^1^Shows data for the 27 who provided baseline QoL and completed daily clinical sign assessments.
